# Survival Time Disparities after Palliative Care Use Among Low-Income Patients on Social Welfare Programs: A Retrospective Cohort Study

**DOI:** 10.1089/pmr.2023.0077

**Published:** 2024-05-15

**Authors:** Daisuke Nishioka, Iku Kanzaki, Ayumi Kihara

**Affiliations:** ^1^ Department of Medical Statistics, Research & Development Center, Osaka Medical and Pharmaceutical University, Takatsuki, Japan.; ^2^ Department of Social Epidemiology, Graduate School of Medicine and School of Public Health, Kyoto University, Kyoto, Japan.; ^3^ Department of Patient Support Center, Kyoto Min-iren Asukai Hospital, Kyoto, Japan.; ^4^ Department of Palliative Care, Kyoto Min-iren Asukai Hospital, Kyoto, Japan.

**Keywords:** observational study, poverty, public assistance, social welfare, survival analysis

## Abstract

**Background:**

Relieving the total pain of patients with cancer and supporting their well-being throughout their lives are important roles of palliative and supportive care. Poverty may inhibit patients from receiving dignified end-of-life care; however, using social welfare services may reduce its impact on patients’ end-of-life experiences. Nevertheless, no study has investigated which social welfare service could lead to favorable end-of-life experiences for patients living in poverty.

**Objective:**

This study aimed to describe the characteristics of users of palliative care among low-income patients and examine the difference in survival time among patients with cancer on social welfare services in a single center in Kyoto, Japan.

**Design:**

We conducted a retrospective cohort study.

**Setting/Subjects:**

We included 220 patients using Public Assistance (PA: aid minimum income and medical/long-term care), Free/Low-Cost Medical Care (FLCMC: aid only medical care), and nonwelfare-users who newly received palliative care in 2021.

**Measurements:**

We calculated patients’ survival time from the initiation of palliative care to death. In addition, we identified patients who experienced home death.

**Results:**

Compared with nonusers, FLCMC beneficiaries had shorter survival times (adjusted hazard ratio [aHR] 2.05, 95% confidence interval [CI] 0.80–5.22). No difference was observed among PA beneficiaries (aHR 1.19, 95% CI 0.49–2.87). No home death was observed among welfare service recipients.

**Conclusions:**

Social welfare benefits only for medical expenses may not sufficiently support dignified end-of-life care for low-income patients. Further studies are required to examine the robustness of this study considering various bio-psycho-social factors that can influence these findings, to support low-income patients with cancer on social welfare services.

## Introduction

Relieving the total pain of patients with cancer and supporting their well-being throughout their lives are important roles of palliative and supportive care. Socioeconomic status is a well-established determinant of people’s health and health behavior.^1^ For example, individuals with poverty face challenges in maintaining healthy lives as they have difficulties in accessing health care,^2^ including palliative care.^3^ Impoverished populations typically experience multidimensional difficulties related to poverty, including not only low income but also psychosocial problems, such as a lack of social support,^4–6^ resulting in disparities in the quality of palliative care among people in low socioeconomic positions.^3,7^

To address multidimensional difficulties among impoverished population, governments in developed countries have implemented several welfare programs that can provide financial support to improve livelihoods and health care access. In Japan, there are two well-known welfare services called Public Assistance (PA; “seikatsu-hogo”) and Free/Low-Cost Medical Care (FLCMC; “muryo-teigaku-shinryo”). PA is a governmental program for people who are living below the poverty line and without any assets. Approximately 1.7% of the Japanese population is enrolled in the PA program.^8^ PA recipients benefit from financial assistance, including minimum income support and access to medical/long-term care services provided by the residential municipality. Meanwhile, the FLCMC program is a voluntary program provided by designated health care institutions.^9^ FLCMC applicants are screened individually at their corresponding institution, and eligible recipients are exempted from out-of-pocket medical payments at the designated institutions. Thus, PA recipients can be regarded as impoverished individuals benefiting from minimum income protection and livelihood, including medical and long-term care costs, whereas FLCMC recipients can be considered as financially constrained individuals benefiting only from medical care costs.

Previous global studies have shown that recipients of social welfare services have more unfavorable health conditions than those of general populations.^10^ For example, PA recipients are more likely to have diabetes,^11^ depressive symptoms,^12^ and suicidal ideation/behavior.^13^ Similarly, FLCMC recipients in Japan are more likely to have lower health-related quality of life, both physically and mentally.^14,15^ Therefore, patients with cancer using social welfare services who receive palliative care may experience more physical and mental problems than those of the patients who are not using social welfare services. However, no real-world evidence exists regarding the availing statuses of social welfare, characteristics, and life course after palliative care use.

Hence, this study aimed to describe the characteristics of users of palliative care among low-income patients on PA and FLCMC, as well as those not benefiting from social welfare services, and compare the survival time across the availing statuses of social welfare services in a single hospital providing palliative care and homecare in Kyoto, Japan.

## Materials and Methods

### Design of the study

We conducted a retrospective cohort study.

### Data

We collected data from adult beneficiaries of the FLCMC and PA programs, as well as from individuals who were not using social welfare services but newly used palliative care at a hospital in Kyoto, Japan, during the fiscal year 2021. We selected this period because of the smallest fluctuations in patient demographics due to social influences during the COVID-19 pandemic. The quality of end-of-life care was affected by the COVID-19 pandemic from the perspectives of public health or politics in the years before and after.

### Participants

We include all patients with cancer who newly used palliative care. In the Japanese medical systems, palliative care units are permitted only for cancer care and partly for acquired immunodeficiency syndrome (AIDS).^16^ Overall, 220 patients with cancer (without AIDS) were included and followed up for one year. Among them, 17 patients were using PA, 8 were using FLCMC, and 195 were not using any welfare services.

### Data collection

All patients who newly visited Asukai hospital for palliative care had opportunities for consultation with physicians, nurses, and social workers. Physicians and nurses collected the following data from the patients: date of cancer diagnosis, cancer types, cancer stages at diagnosis, and survival time from the first visits to palliative care. Social workers collected data on patients’ sociodemographic backgrounds, including their utilization of social welfare supports and living arrangements.

### Variables

#### Outcome measure

The primary outcome measure was overall survival, defined as the interval from the date of the first visits for palliative care to the date of death due to any cause. To identify censored or deceased patients, vital status (alive or dead) at the date of last contact was used. We estimated follow-up time capped at 30;days to identify the patients who needed rapid establishment of their support system after the initial visit, and all surviving patients were censored after the 30th day.

The second outcome measure was events related to patients’ home death. Mortality may not be the best measure of the quality of death among patients with cancer. In Japan, “home” is the most preferred place among patients with cancer to pass away,^17^ signifying high-quality end-of-life care. Therefore, we included this as the second outcome, considering it as a marker of good quality end-of-life care experience among patients with cancer.^18^

#### Explanatory variables

The explanatory variables were the availing statuses of welfare services (FLCMC, PA, and not using).

#### Covariates

We considered sex (male/female), age at the initial visit, cancer stages at their cancer diagnoses, living arrangement (living alone or not), and the existence of a caregiver as a proxy measure of social support for the patients (none or someone including spouse, parents, siblings, children) as covariates.

### Statistical analysis

First, we described the characteristics of the study participants. Second, the Kaplan-Meier analysis was performed to compare overall survival based on the patient’s availing status of social welfare services. Multivariable Cox regression analysis was performed to evaluate the association between survival outcomes and social welfare service use. Model 1 was a crude model that included only the explanatory variables. Model 2 was adjusted for age and sex to account for sociodemographic distribution across explanatory variables. Model 3 was further adjusted for living because the state of living alone can predict survival time. Finally, Model 4 included stages of cancer at diagnosis as a potential confounder. As an additional analysis to eliminate the bias due to cancer progression and the place of receiving palliative care as confounders, we analyzed Model 4, restricting the analytical sample to patients with stage IV cancer and included the variable of the place of receiving palliative care (home-based/hospital-based). An alpha level of 0.05 was considered significant; all tests were two-tailed. All statistical analyses were performed using STATA MP Ver.18 (StataCorp, College Station, TX).

### Ethical considerations

The institutional ethics committee approved this study’s protocol (Approval No: 2022-10-3).

## Results

Among the study participants, 195 were not using any welfare support, 17 were using PA, and 8 were using FLCMC. During the one-year follow-up time, we identified 178 deaths, which included 84 deaths on the first 30th day. The proportion of the events that occurred on the 30th day was higher among beneficiaries of FLCMC (five events, 62.5%) and PA (seven events, 41.2%) than among those no using welfare services (72 events, 36.9%) (Table 1).

**Table 1.** Patients’ Sociodemographic Characteristics across Availing Statuses of Social Welfare Services (*n* = 220)

		Availing status of social welfare					
		Not using		Public Assistance		Free/Low-Cost Medical Care	
Character	Category	*n* = 195	%	*n* = 17	%	*n* = 8	%
Sex							
	Female	84	43.1	5	29.4	1	12.5
	Male	111	56.9	12	70.6	7	87.5
Age							
	Mean, SD	77.1, 11.4		76.3, 11.0		79.3, 12.0	
Living arrangement							
	Living with someone	147	75.4	2	11.8	2	25.0
	Living alone	48	24.6	15	88.2	6	75.0
Cancer stage at diagnosis							
	I, II	19	9.7	0	0.0	0	0
	III	28	14.4	1	5.9	1	12.5
	IV	98	50.3	11	64.7	5	62.5
	Unknown	50	25.6	5	29.4	2	25

SD, standard deviation.

**FIG. 1.**
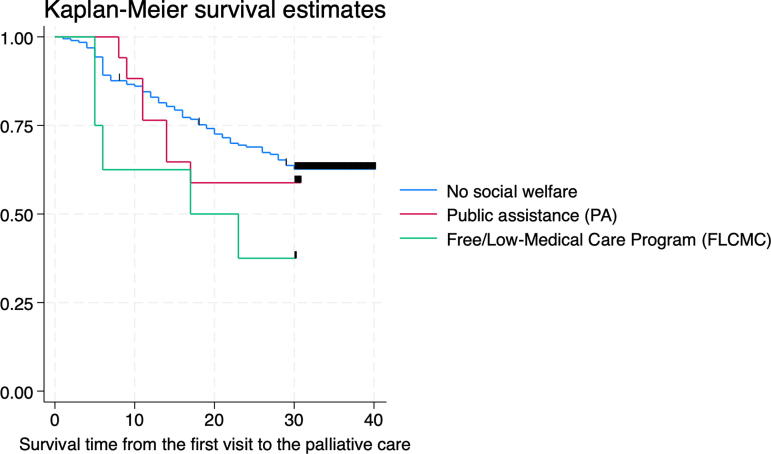
Kaplan-Meier survival estimates from the first visit to the palliative care across social welfare services.

The Kaplan-Meier analysis showed that using social welfare services, especially FLCMC, led to a lower survival rate (Fig. 1). The results of univariable Cox regression analysis (Model 1) showed that the hazard ratios (HRs) were 2.22 (95% confidence interval [CI] 0.90–5.50) and 1.18 (95% CI 0.54–2.56) among FLCMC and PA beneficiaries, respectively. Fully adjusted multivariable Cox regression analysis showed that the adjusted HR (aHR) was 2.05 (95% CI 0.80–5.22) and 1.19 (95% CI 0.49–2.87) among FLCMC and PA beneficiaries, respectively (Table 2). Because no observations existed where cancer stage at diagnosis was I or II among FLCMC and PA users, the additional analysis restricting the analytical sample to patients with stage IV cancer showed aHR of 2.80 (95% CI 0.87–8.95) and 1.82 (95% CI 0.61–5.44) among FLCMC and PA beneficiaries, respectively (Supplementary Table S1). In addition, receiving hospital-based palliative care was identified as a predictor of lower survival rates (Supplementary Table S1). Home death was only observed among patients not using social welfare support (23 patients).

**Table 2.** Adjusted Hazard Ratio of Survival Rate of Patients Receiving Palliative Care across Different Social Welfare Services

	Model 1			Model 2			Model 3			Model 4		
	HR	95% CI		aHR	95% CI		aHR	95% CI		aHR	95% CI	
Explanatory variable												
Social welfare												
Not using	Ref			Ref			Ref			Ref		
Public assistance	1.18	0.54	2.56	1.17	0.54	2.55	1.18	0.49	2.86	1.19	0.49	2.87
Free/Low-Cost Medical Care	2.22	0.90	5.50	2.14	0.86	5.33	2.05	0.81	5.19	2.05	0.80	5.22
Covariates												
Age												
By one year				1.02	1.00	1.04	1.02	0.99	1.04	1.02	1.00	1.04
Sex												
Female				Ref			Ref			Ref		
Male				1.14	0.72	1.79	1.14	0.72	1.81	1.13	0.71	1.78
Living arrangement												
Living with someone							Ref			Ref		
Living alone							1.36	0.82	2.28	1.34	0.80	2.24
Existence of Caregivers												
No							Ref			Ref		
Yes							1.69	0.76	3.78	1.73	0.76	3.91
Cancer stage at diagnoses												
I, II										Ref		
III										1.94	0.63	5.96
IV										2.05	0.73	5.72
Unknown										1.30	0.43	3.89

aHR, adjusted hazard ratio; CI, confidence interval; Ref, reference category of the explanatory variable used in the regression analyses.

## Discussion

### Main findings of this study

In the present study, we observed that men are more likely to use social welfare services than women and patients using social welfare services are diagnosed with cancer at later stages, consistent with previous research.^19^ Our study revealed that patients receiving FLCMC experienced lower survival rates than those of patients receiving PA and the comparison group. No home death was observed among social welfare services recipients, which is consistent with previous research.^7^

### What this study adds

The lower survival rate among FLCMC beneficiaries observed in our study may be attributed to the multidimensional difficulties faced by poor patients in their daily lives, as suggested in previous studies.^7^ Impoverished individuals face not only financial challenges in accessing medical care but also other aspects of poverty, including livelihood difficulties, less social support, and time constraints in which individuals do not have sufficient discretionary time.^4–6^ Although FLCMC only covers medical expenses at the designated hospitals, PA recipients benefit from their monthly minimum income support and access to health care services, including visiting nurses, medications, and long-term care services and supports. Thus, providing sufficient support, including nursing or long-term care services, could potentially improve health and well-being among low-income patients with cancer receiving palliative care because no difference in survival rate was observed between PA recipients and individuals not using social welfare services. Notably, social welfare benefits only for medical expenses might not be sufficient to support dignified end-of-life care among poor patients.

### Strengths and limitations of the study

Although this was the first study investigating the usage of palliative care among low-income patients with cancer on social welfare services, it had limitations due to its single-center and retrospective design, focusing on a marginalized population in poverty during the COVID-19 pandemic. First, we could only use the small sample obtained from a single institution in the year, resulting in a lack of statistical power. Therefore, a future study recruiting more participants over a longer period will be essential. Second, the income level of the comparison group was not considered because of the retrospective nature of the data, as hospitals/clinics do not collect patients’ information on income in usual medical care, especially for people not applying for social welfare services. Most patients not using social welfare services have higher income and better health conditions, and this may have overestimated the effect of FLCMC. Third, although we included the caregiver variable to account for the impact of social support on patients, we were unable to consider psychosocial factors related to multidimensional poverty. Fourth, we could only include patients with cancer who used palliative care due to constraints within the medical systems. Future analyses should encompass patients with other etiologies. Fifth, we only used the time and place of death as outcome variables; however, to identify the dignity of end-of-life care among patients with cancer, considering patients’ experiences and quality of lives is important. Finally, generalizability was limited because this study used data from a single health care institution in Kyoto. Considering these limitations, future prospective studies focusing on recruiting more patients, including those with similar incomes who are not enrolled in FLCMC or PA programs, being conducted across multiple institutions, and collecting broader bio-psycho-social factors including patients’ preferences and experiences are essential.

## Conclusion

Although our results were not statistically significant, they revealed that low-income patients using FLCMC may have lower survival rates and home death than those of individuals using PA or not using social welfare services. Social welfare benefits only covering medical expenses may not be sufficient to support dignified end-of-life care among low-income patients. Due to the retrospective and single-center nature of our research, further studies are required to examine the robustness of this study, including broader bio-psycho-social factors that can influence the outcomes, to support low-income patients with cancer receiving social welfare services.

## Research Ethics and Patient Consent

This study protocol was approved by the Ethics Committee of the Kyoto Min-iren Asukai Hospital (Approval No: 2022-10-3). All methods were performed in accordance with relevant guidelines and regulations of the Japanese government.

## Data Sharing Statement

The data used in this study were collected from patients of Kyoto Miniren Asukai Hospital, which are not publicly available. The data are available from the authors upon reasonable request, with the permission of the facility. Please contact the corresponding author for the request.

## Supplementary Material

Supplementary Table S1

## Abbreviations Used

AIDS: Acquired immunodeficiency syndrome
CI: Confidence interval
FLCMC: Free/low-cost medical care
HR: Hazard ratio
PA: Public assistance
