# Supplementary material for: Survival Time Disparities after Palliative Care Use Among Low-Income Patients on Social Welfare Programs: A Retrospective Cohort Study
Source: Palliat Med Rep. 2024 May 15;5(1):187–93. doi: 10.1089/pmr.2023.0077 (PMC11265612; doi:10.1089/pmr.2023.0077)
Supplement: Supplementary Table S1 [file pmr.2023.0077_supplemental_file.docx]

Supplementary Table S1. Adjusted hazard ratio (aHR) of survival rate: a sensitivity analysis restricting the analytical sample to patients with stage IV cancer (N=113)

|  |  |  |  | HR | 95% CI |  |
| --- | --- | --- | --- | --- | --- | --- |
| *Explanatory variable* | Social welfare | |  |  |  |  |
|  |  | Not using |  | Ref |  |  |
|  |  | Public assistance |  | 1.82 | 0.61 | 5.44 |
|  |  | Free Low-Cost Medical Care |  | 2.80 | 0.87 | 8.95 |
| *Covariates* | Age |  |  |  |  |  |
|  |  | by 1 year |  | 1.02 | 0.99 | 1.04 |
|  | Sex |  |  |  |  |  |
|  |  | Female |  | Ref |  |  |
|  |  | Male |  | 0.89 | 0.48 | 1.66 |
|  | Living arrangement | |  |  |  |  |
|  |  | Living with someone |  | Ref |  |  |
|  |  | Living alone |  | 0.57 | 0.27 | 1.22 |
|  | Existence of Caregivers | |  |  |  |  |
|  |  | No |  | Ref |  |  |
|  |  | Yes |  | 1.37 | 0.46 | 4.07 |
|  | Place started to receive palliative care | | | |  |  |
|  |  | Home |  | Ref |  |  |
|  |  | Hospitals |  | 3.72 | 1.93 | 7.16 |

aHR, adjusted hazard ratio; CI, confidence interval; HR, hazard ratio
